# Dietary Supplementation of *Bacillus* sp. PM8313 with β-glucan Modulates the Intestinal Microbiota of Red Sea Bream (*Pagrus major*) to Increase Growth, Immunity, and Disease Resistance

**DOI:** 10.3389/fimmu.2022.960554

**Published:** 2022-07-22

**Authors:** Won Je Jang, Mi-Hyeon Jeon, Su-Jeong Lee, So Young Park, Young-Sun Lee, Da-In Noh, Sang Woo Hur, Seunghan Lee, Bong-Joo Lee, Jong Min Lee, Kang-Woong Kim, Eun-Woo Lee, Md Tawheed Hasan

**Affiliations:** ^1^ Department of Biotechnology, Pukyong National University, Busan, South Korea; ^2^ Biopharmaceutical Engineering Major, Division of Applied Bioengineering, Dong-Eui University, Busan, South Korea; ^3^ Aquafeed Research Center, National Institute of Fisheries Science, Pohang, South Korea; ^4^ Department of Smart Fisheries Resources, College of Industrial Sciences, Kongju National University, Yesan, South Korea; ^5^ Core-Facility Center for Tissue Regeneration, Dong-Eui University, Busan, South Korea; ^6^ Department of Aquaculture, Sylhet Agricultural University, Sylhet, Bangladesh

**Keywords:** digestive enzymes, immunity, microbiota, pagrus major, red sea bream

## Abstract

A 56-day feeding trial was conducted to determine the effect of dietary supplementation with *Bacillus* sp. isolated from the intestines of red sea bream on the growth performance, immunity, and gut microbiome composition of red sea bream. Three diets (a control diet and two treatments) were formulated without *Bacillus* sp. PM8313 or β-glucan (control, CD), 1 × 10^8^ CFU g^−1^ PM8313 (BSD), and 1 × 10^8^ CFU g^−1^ PM8313 + 0.1% β-glucan (BGSD). At the end of the experiment, the weight, specific growth rate, feed conversion ratio, and protein efficiency ratio of the fish in the BSD and BGSD diet groups were significantly improved than those of the control group (P < 0.05). Additionally, amylase and trypsin activities were significantly higher (P < 0.05) in both groups compared to the control. Superoxide dismutase and lysozyme activity, which are serum non-specific immune responses, only increased in the BGSD group. The two treatment groups exhibited a marked difference in the intestinal microbiota composition compared to the control group. Furthermore, the treatment groups exhibited an upregulation of IL-6 and NF-κb, coupled with high survival rates when challenged with *Edwardsiella tarda*. Therefore, dietary supplementation with PM8313 improved the growth performance, digestive enzyme activity, non-specific immunity, and pathogen resistance of red sea bream, in addition to affecting the composition of its intestinal microflora.

## Introduction

Red seabream (*Pagrus major*) is one of the most prominent cultured finfish species in East-Asian countries such as China, Japan, and the Korean peninsula (1). The artificial breeding of this species for aquaculture production began in 1980 in Korea. In 2020, this species became the 4th most important aquaculture species in the Republic of Korea, with annual production yields reaching 5,800 tonnes, which constitutes approximately 6.6% of the total national finfish production (88,200 tons) (2). In Korea, sea bream culture has recently intensified due to the availability of fingerlings, the pleasant flavor and palatability of its meat, high market demand and economic turnover, and government research investment.

Nevertheless, the intensification of red sea bream aquaculture has also led to widespread concerns regarding infectious disease outbreaks in culture systems, which have resulted in substantial economic losses. Intensive culture demands a high supply of artificial feed, of which a certain portion remains uneaten, in addition to limited water exchange, high stocking density, and application of growth promoters, all of which have been linked to environmental degradation (3, 4). Poor aquatic environments weaken the immune system of fish, which makes them more vulnerable to pathogenic and opportunistic bacteria in culture farms. Particularly, *Edwardsiella tarda*, *Vibrio alginolyticus*, and *Lactococcus garvieae* (5) are among the most common pathogens in *P. major*, resulting in hemorrhage, exophthalmia, skin lesions and ulcers, erratic swimming, nervous dysfunction, and sudden death, all of which have led to massive economic losses. To control diseases, farmers apply synthetic antibiotics and chemotherapeutics in the feed and water, and these practices are often conducted in an unscientific manner.

In addition to eliminating pathogens, antibiotics also non-specifically eliminate beneficial aquatic bacteria in the environment, remain in the fish muscles, and promote antibiotic resistance, thereby posing a serious human health threat (6). In fact, antibiotic-resistant pathogens have previously been identified in both fish and humans (7). To overcome the aforementioned challenges, some countries have already banned antibiotics in aquaculture and scientists are actively searching for eco-friendly alternatives, among which probiotics are considered a promising candidate treatment (8).

The World Health Organization and Food and Agricultural Organization (9) defined probiotics as “a live microorganism administrated at an appropriate concentration that exerts beneficial effects on host health and immune parameters.” The action mechanisms of probiotics are commonly categorized as antagonistic (e.g., nisin and bacteriocin), sources of nutrients and digestive enzymes, adhesion and colonization of the gastrointestinal tract (GIT) for pathogen exclusion, and upregulation of immunity and immune-related gene transcription (10, 11). Autochthonous bacteria that inhabit the mucosal layer of the intestine of aquatic animals are an excellent basis for the development of aquaculture probiotics (Van 12). Moreover, host-associated intestinal probiotics are more likely to survive and colonize the harsh environment of the GIT, thereby promoting fish health by upregulating immune-related genes and controlling infectious diseases.

Due to the many benefits of probiotics, there are ongoing efforts to identify and characterize novel beneficial bacterial strains in fish intestines, as well as to assess the benefits of these probiotics through dietary supplementation. Previous studies have assessed the effects of dietary supplementation with different probiotics [e.g., heat-killed *Lactobacillus plantarum* (13) and *Pediococcus pentosaceus* (14), *L. rhamnosus* (15) and/or *L. lactis* (16), *Bacillus subtilis* (17), and *B. subtilis* C-3102 (18)] in *P. major*. These probiotics, some of which were commercial or derived from other sources, were found to improve growth, feed utilization, immunomodulation, antioxidant activity, serum biochemistry, and disease resistance. However, none of them was isolated from red sea bream. Therefore, to the best of our knowledge, our study is the first to assess the dietary administration of *Bacillus* sp. PM8313 isolated from the intestinal tract of *P. major*, as well as the probiotic effects of this treatment in red sea bream. Moreover, *in vitro* characterization of strain PM8313 demonstrated that this bacterium possesses probiotic potential, and higher utilization of β-glucan for its growth and survival compared to fructooligosaccharides, mannan oligosaccharide, and inulin. β-glucan is a widely recognized immune stimulant that also possesses prebiotic (low-density oligosaccharide) potential (19, 20) for the modulation of growth, immunity, and disease resistance in *Salmo salar* (21), *Oreochromis niloticus* (22), *Cyprinus carpio* (23), and many commercial fish species. Therefore, our study not only sought to assess the probiotic potential of strain PM8313, but also its ability to ferment β-glucan inside the intestinal environment to improve innate immunity, beneficial bacterial richness in the intestine, and disease resistance in *P. major*.

Specifically, the objectives of this study were to characterize the effectiveness of host-associated *Bacillus* sp. PM8313 both alone and paired with β-glucan to enhance *P. major* growth, innate immunity, and edwardsiellosis resistance. Moreover, digestive enzyme activities, immune gene transcription, and intestinal microbial community modulation were also quantified to assess the effectiveness of this newly isolated probiotic.

## Materials and Methods

All animal procedures were performed in accordance with the National Research Council guidelines (Guide for the Care and Use of Laboratory Animals) and with approval from the Dong-eui University Laboratory Animal Ethics Committee.

### Experimental Diet Preparation

*Bacillus* sp. PM8313 (KCTC14892BP), which was used as a dietary supplement in this study, was isolated from the intestine of wild red sea bream and identified by 16S rRNA sequencing analysis (**Supplementary Figure 1**). Three types of feed were used to investigate the effects of PM8313 supplementation. The composition of the control diet (CD) is shown in **Table 1**. After measuring the required amount of ingredients, 300 ml/kg of distilled water and fish oil were added and mixed thoroughly to prepare CD. A bacterial supplement diet (BSD) was formulated by adding PM8313 to that 300 ml of water to adjust a bacterial concentration at 3.34 × 10^8^ CFU/ml and mixed with CD ingredients to ensure PM8313 at 1 × 10^8^ CFU/g diet. In the bacterial and β-glucan supplementation diet (BGSD), 0.1% cellulose of CD was replaced by 0.1% β-glucan (24), and then followed the B SD preparation protocol. Without any heat production, feeds were prepared by a pelleting machine (Baokyong, South Korea), air-dried at room temperature, and stored at −4°C in a sealed polybag. Feed proximate composition analysis was performed in accordance with the AOAC (25).

**Table 1:** Composition of the basal experimental diet for red sea bream (*Pagrus major*).

Ingredients	Percentage (%)	Feed proximate composition	Percentage (%)
Fish meal	30.00	Moisture	5.02
Wheat flour	28.88	Crude protein	44.02
Chicken by product	31.20	Crude lipid	13.78
Fish oil	4.00	Crude ash	12.61
Squid liver powder	3.00		
Lecithin	1.00	Supplement for BSD group	
Mono calcium phosphate	0.20	*Bacillus* sp. PM8313	1 × 10^8^ CFU/g
Vitamin C	0.50		
Vitamin premix^a^	0.50	Supplements for BGSD group	
Mineral premix^b^	0.50	*Bacillus* sp. PM8313	1 × 10^8^ CFU/g
Choline^c^	0.12	β-glucan	0.10%
Cellulose	0.10		

^a^Vitamin premix (as mg kg−1 in diets): Ascorbic acid, 300; dl-Calcium pantothenate, 150; Choline bitate, 3000; Inositol, 150; Menadion, 6; Niacin, 150; Pyridoxine. HCl, 15; Rivoflavin, 30; Thiamine mononitrate, 15; dl-α-Tocopherol acetate, 201; Retinyl acetate, 6; Biotin, 1.5; Folic acid, 5.4; Cobalamin, 0.06. ^b^Mineral premix (as mg kg−1 in diets): NaCl, 437.4; MgSO4·7H2O, 1379.8; ZnSO4·7H2O, 226.4; Fe-Citrate, 299; MnSO4, 0.016; FeSO4, 0.0378; CuSO4, 0.00033; Ca(IO)3, 0.0006; MgO, 0.00135; NaSeO3, 0.00025.

### Fish Maintenance and Feeding Trial

Red sea bream was obtained from the Nam-Bu fish farm (Yeosu, Republic of Korea) and divided into 360 L semi-recirculating tanks to acclimatize for 1 week. After acclimatization, 180 healthy red seabreams were randomly assigned to three groups. The fish were fed twice a day at 9:00 and 16:00 until apparent satiation. Water quality was regularly monitored, and stable environmental parameters were maintained (temperature, 18.0°C ± 0.5°C; salinity, 32.3 ± 0.7 ppt; dissolved oxygen, 5.6 ± 0.3 mg/L; pH, 7.8 ± 0.2; water flow, 1.2 L/min).

### Growth Performance, Feed Utilization, and Body Indices

After 8 weeks of the feeding trial, growth performance, feed utilization, and organosomatic indices were calculated as follows:

• Weight gain (WG; %) = 100 × (Final weight − Initial weight)/Initial weight

• Specific growth rate (SGR; %/day) = 100 × (ln final weight − ln initial weight)/days

• Feed conversion ratio (FCR) = Dry feed intake/Wet body WG

• Protein efficiency ratio (PER) = Wet body WG/Protein fed

• Condition factor (CF; %) = 100 × Body weight/(Total body length)^3^

• Viscerosomatic index (VSI; %) = 100 × Visceral weight/Body weight

• Hepatosomatic index (HSI; %) = 100 × Liver weight/Body weight

### Analysis of Digestive Enzymes

Digestive enzyme activity on the anterior midgut of fish (n = 5) from each group was analyzed using amylase, trypsin, and lipase activity assay kits (BioVision, USA) according to the manufacturer’s instructions.

### Nonspecific Immune Parameter Analysis

Superoxide dismutase (SOD), lysozyme, and myeloperoxidase (MPO) activities in serum were assessed using an SOD activity colorimetric assay kit (BioVision), a lysozyme detection kit (Sigma-Aldrich), and an MPO colorimetric assay kit (Sigma-Aldrich), respectively, according to the manufacturers’ instructions.

### Serum Biochemical Parameter Analysis

The levels of serum biochemical parameters such as serum alanine aminotransferase (ALT), aspartate aminotransferase (AST), total glucose, and total cholesterol were measured using Mindray commercial kits and a Mindray BS-390 automatic biochemistry analyzer (Mindray Bio-Medical Electronics, China) at the Core-Facility Center of Dong-eui University (Busan, South Korea).

### Intestinal Microbiota Analysis

Total microbial DNA was isolated from the intestines of sea bream fed with the experimental diets for 8 weeks using the FavorPrepTM Tissue Genomic DNA Extraction Mini Kit (Favorgen Biotech Corp., Taiwan). The quality of the total DNA was assessed through gel electrophoresis and the V3-V4 region was amplified to construct a library. The prepared library was sequenced on an Illumina MiSeq system (300 bp paired-end reads).

### Gene Expression Analysis

Real-time quantitative polymerase chain reaction (RT-qPCR) was performed to investigate immune-related gene expression. A Hybrid-R RNA purification kit and Riboclear plus kit (GeneAll Biotechnology, South Korea) were used for RNA isolation and residual DNA removal from red sea bream intestines. Next, cDNA was synthesized from the isolated RNA using the PrimeScript 1st strand cDNA synthesis kit (Takara, Japan). Gene expression was examined using TB Green Premix Ex Taq (Takara, Japan) on a TP700/760 Thermal Cycler Dice Real Time System (Takara, Japan), and relative expression was calculated using the Thermal Cycler Dice software V5.0× with the 2^-ΔΔCT^ method and β-actin as a reference gene. The gene-specific primers used for gene amplification are summarized in **Table 2**.

**Table 2:** Gene-specific primers used to quantify relative gene expression.

Gene	Oligonucleotide Sequence (5` to 3`)	
	Forward	Reverse
β-actin	CAAAGCCAACAGGGAGAAG	TACGACCAGAGGCATACAG
IL-6	ACAACATCCCCTCACTTCC	CCTCTTTCTCCACATACTTCAG
IL-8	AGGACAGGCCAAGAGGTTTG	AGTGTGTTTGGGTGCCCTTA
NF-κB	ACACTCTTCCTACAGCAGCG	TCCTCCATAACCCAACCCAC
TNF-α	ATCAGCAGCAAAGCCAAG	GTTGTCAACCAGTCGGAAG
HSP70	GGACATCAGCGACAACAAG	CGGAAGAGGTCAGCATTGAG
GH	ACCAGAACCAGAACCAGAAC	CAGACAGAGAGAGAGAGAGAG
TLR	TCATCATCAGCAACAACCAG	TCAGGAGGCAAATAGGAGAG

### *Edwardsiella tarda* Challenge Experiments

The pathogenic bacterium *E. tarda* (ATCC 15947) was purchased from the Korean Collection for Type Cultures (Seoul, South Korea). *E. tarda* was cultured in brain heart infusion (BHI) broth at 30°C and washed three times with phosphate-buffered saline. Five fish from each tank (n = 15 fish per group) were randomly collected and anesthetized using 2-phenoxyethanol. The fish were intraperitoneally injected with 100 μL (1 × 10^8^ CFU/mL) of E. tarda. Fish mortality in each tank was monitored every 6 h up to 100% death in the control group, and swabs from tissue samples were collected and spread on a BHI agar plate to confirm edwardsiellosis.

### Statistical Analysis

The statistical significance of the data was analyzed by one-way analysis of variance using SPSS (IBM, USA), followed by Duncan’s multiple range test.

## Results

### Growth Performance, Feed Utilization, and Body Indices

Dietary administration with BSD or BGSD significantly (*P* < 0.05) enhanced WG, SGR, FCR, and FCR compared with CD (**Table 3**). However, there were no significant differences in any of the evaluated parameters between the BSD and BGSD groups. Furthermore, the body indices (CF, VSI, and HSI; **Table 3**) and whole-body proximate composition (data not shown) of red sea bream did not vary significantly between the BSD or BGSD groups after 8 weeks of the feeding trial. These results demonstrated that oral administration of both PM8313 and PM8313 + β-glucan enhanced the growth and feed utilization of red sea bream.

**Table 3:** Growth performance, feed utilization, and organosomatic indices of red sea bream supplemented with the experimental feed additives.

Groups	Growth performance, feed utilization and organosomatic parameters						
	WG (%)	SGR (% day^-1^)	FCR	PER	CF (%)	VSI (%)	HSI (%)
CD	120.84 ± 3.48^a^	1.41 ± 0.03^a^	1.46 ± 0.05^b^	1.43 ± 0.05^a^	1.70 ± 0.06	2.07 ± 0.10	2.20 ± 0.04
BSD	131.41 ± 4.15^b^	1.50 ± 0.03^b^	1.23 ± 0.04^a^	1.69 ± 0.05^b^	1.73 ± 0.15	2.08 ± 0.11	2.19 ± 0.07
BGSD	134.61 ± 4.55^b^	1.52 ± 0.03^b^	1.33 ± 0.06^a^	1.57 ± 0.07^b^	1.77 ± 0.07	2.09 ± 0.13	2.16 ± 0.07

Values are mean ± SD of three replicates. Values with different superscript letters within the same column in the table are significantly different (P < .05). The lack of superscript letter indicates no significant differences (P > 0.05). Control diet, CD: without Bacillus sp. PM8313 or β-glucan, BSD: 1 × 10^8^ CFU g^−1^ Bacillus sp. PM8313, and BGSD: BSD + 0.1% β-glucan.

### Analysis of Digestive Enzymes

**Figure 1:**
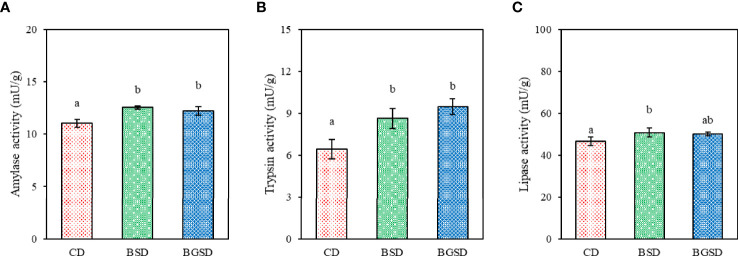
Comparison of the measured amylase **(A)**, trypsin **(B)**, and lipase **(C)** activities between the three groups. The data represent the mean ± standard deviation; different letters indicate statistically significant differences between groups (*P* < 0.05). Control diet, CD: without *Bacillus* sp. PM8313 or β-glucan, BSD: 1 × 10^8^ CFU g^−1^ *Bacillus* sp. PM8313, and BGSD: BSD + 0.1% β-glucan.

The amylase and trypsin activities were significantly increased in the BSD (12.56 ± 0.14 and 8.63 ± 0.71, respectively) and BGSD (12.22 ± 0.41 and 9.47 ± 0.55, respectively) groups compared to the CD (11.03 ± 0.37 and 6.42 ± 0.68, respectively) group. Significant differences in lipase activity occurred only between the BSD (50.71 ± 2.16) and CD (46.66 ± 2.05) groups. There were no significant differences between the BSD and BGSD groups in any of the enzyme assays conducted herein (**Figure 1**).

### Nonspecific Immune and Serum Biochemical Parameter Analysis

**Figure 2:**
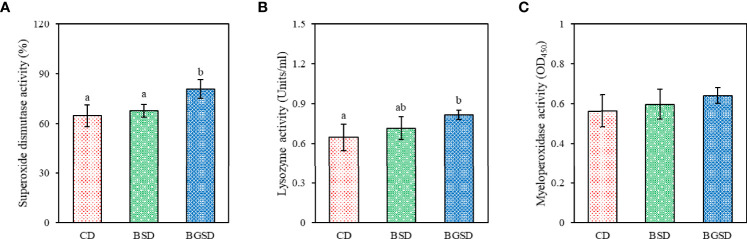
Serum non-specific immune parameters [**(A)**, superoxide dismutase; **(B)**, lysozyme activity; **(C)**, Myeloperoxidase activity] of red sea bream fed with the experimental diets. The data represent the mean ± standard deviation; different letters indicate statistically significant differences between groups (*P* < 0.05). Control diet, CD: without *Bacillus* sp. PM8313 or β-glucan, BSD: 1 × 10^8^ CFU g^−1^ *Bacillus* sp. PM8313, and BGSD: BSD + 0.1% β-glucan.

SOD activity was significantly increased in the BGSD group compared to the other two groups. Significant differences in serum lysozyme activity occurred only between the BGSD (0.82 ± 0.04) and CD (0.65 ± 0.10) groups. There was no significant difference in MPO activity between the groups (**Figure 2**). The investigated serum biochemical parameters (AST, ALT, total glucose, and total cholesterol) were not significantly affected by feed additives (**Table 4**).

**Table 4:** Biochemical parameters of red sea bream fed with the experimental diets.

Groups	Serum biochemical parameters			
	AST (U L^-1^)	ALT (U L^-1^)	Total glucose (mg dl^-1^)	Total cholesterol (mg dl^-1^)
CD	39.33 ± 1.53	12.33 ± 2.52	45.90 ± 2.70	133.83 ± 11.45
BSD	40.33 ± 2.08	12.33 ± 4.16	45.00 ± 5.62	137.50 ± 7.78
BGSD	38.33 ± 5.86	11.33 ± 2.08	46.80 ± 3.12	139.33 ± 8.40

Values are mean ± SD of three replicates. All values within the same column in the table are not significantly different (P > 0.05). Control diet, CD: without Bacillus sp. PM8313 or β-glucan, BSD: 1 × 10^8^ CFU g^−1^ Bacillus sp. PM8313, and BGSD: BSD + 0.1% β-glucan.

### Intestinal Microbiome Analysis

The studied feed additives significantly decreased the ACE, CHAO, and Jackknife alpha diversity estimators. The Shannon index was also significantly decreased in the BSD (2.66 ± 0.20) and BGSD (2.27 ± 0.37) groups compared to the CD (5.14 ± 0.06) group. Significant differences in the Simpson index occurred only between the CD (0.01 ± 0.01) and BGSD (0.26 ± 0.13) groups (**Table 5**). These results demonstrated that the intestinal bacterial community of red sea bream was altered by the feed additives.

**Table 5:** Alpha diversity of the intestinal bacterial communities of red sea bream (*Pagrus major*).

Groups	ACE	CHAO	Jackknife	Shannon	Simpson
CD	1722 ± 83^b^	1670 ± 70^b^	1803 ± 99^b^	5.41 ± 0.06^b^	0.01 ± 0.01^a^
BSD	162 ± 24^a^	157 ± 21^a^	169 ± 23^a^	2.66 ± 0.20^a^	0.12 ± 0.01^ab^
BGSD	295 ± 52^a^	284 ± 50^a^	308 ± 62^a^	2.27 ± 0.37^a^	0.26 ± 0.13^b^

Control diet, CD: without Bacillus sp. PM8313 or β-glucan, BSD: 1 × 10^8^ CFU g^−1^ Bacillus sp. PM8313, and BGSD: BSD + 0.1% β-glucan.

**Figure 3:**
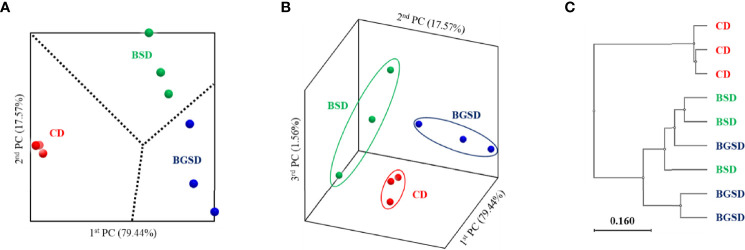
Principal coordinate analysis based on the weighted UniFrac metrics **(A, B)** and unweighted pair group method with arithmetic mean tree **(C)** of bacterial operational taxonomic units between the different diets. Control diet, CD: without *Bacillus* sp. PM8313 or β-glucan, BSD: 1 × 10^8^ CFU g^−1^ *Bacillus* sp. PM8313, and BGSD: BSD + 0.1% β-glucan.

Analysis of the beta-diversity at the genus level based on UniFrac metrics using principal coordinate analysis elucidated clear differences between the feed additive groups and CD group. The BSD and BGSD groups clustered relatively close, and there was no clear difference between the groups in some samples (**Figure 3**).

**Figure 4:**
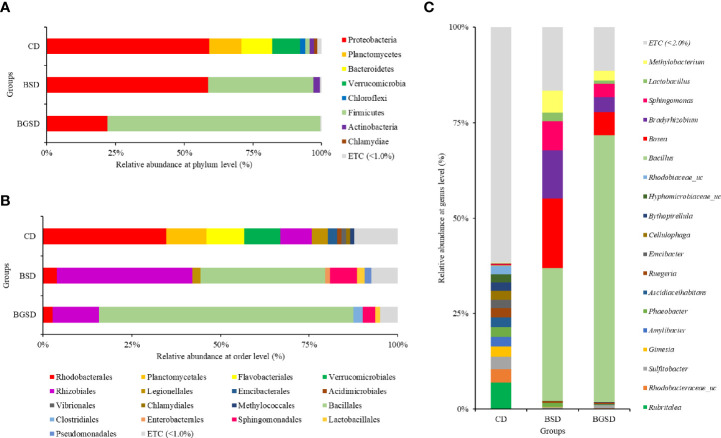
Composition and relative abundance of intestinal bacterial communities of red sea bream with different diets at the phylum **(A)**, order **(B)**, and genus **(C)** level. Control diet, CD: without *Bacillus* sp. PM8313 or β-glucan, BSD: 1 × 10^8^ CFU g^−1^ *Bacillus* sp. PM8313, and BGSD: BSD + 0.1% β-glucan.

**Figure 5:**
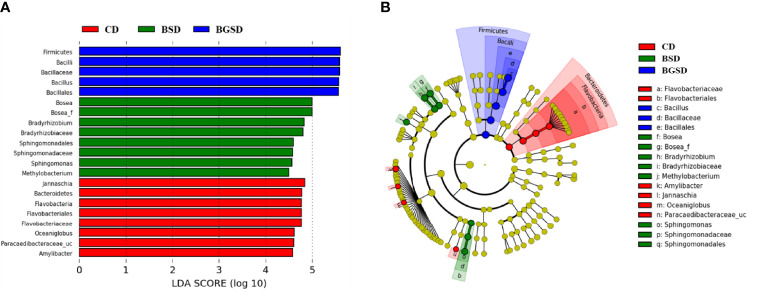
Linear discriminant analysis effect size (LEfSe) analysis of the differential abundance of taxa within red sea bream intestinal microbiota following random sampling from each group. **(A)** Linear discriminant analysis (LDA) score of the abundance of different taxa; **(B)** cladogram showing differentially abundant taxa among the three groups from phylum to genus. Control diet, CD: without *Bacillus* sp. PM8313 or β-glucan, BSD: 1 × 10^8^ CFU g^−1^ *Bacillus* sp. PM8313, and BGSD: BSD + 0.1% β-glucan.

**Figure 6:**
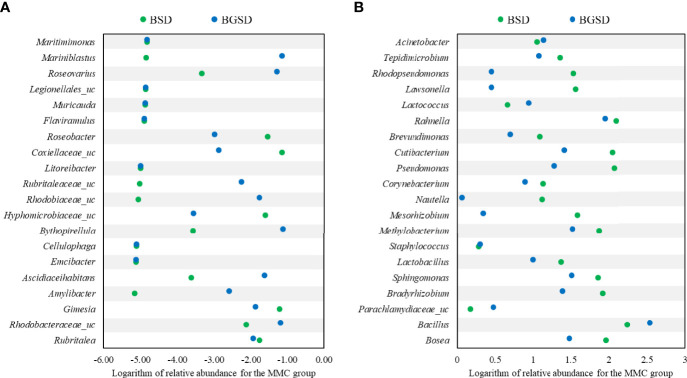
Comparison of the relative abundance of decreased **(A)** or increased **(B)** OTUs compared to the control group. BSD: 1 × 10^8^ CFU g^−1^ *Bacillus* sp. PM8313, and BGSD: BSD + 0.1% β-glucan.

Upon comparing the relative abundances from the phylum to genus levels, our findings indicated that the feed additives significantly affected the composition of the gut microbiota of red sea bream. The largest difference in the relative abundance between groups was observed in the *Bacillus* genus, and the BSD and BGSD groups had higher ratios than the CD group (**Figure 4**). BGSD containing β-glucan as a carbon source for *Bacillus* sp. PM8313 had a higher *Bacillus* abundance and a significantly higher LDA score than BSD (**Figure 5**). The composition ratio of several genera including *Bacillus*, *Bosea*, *Bradyrhizobium*, and *Sphingomonas* was higher in the group supplemented with additives compared to the CD group. In contrast, the ratio of *Cellulophaga*, *Litoreibacter*, *Muricauda*, and *Maritimimonas* decreased (**Figure 6**).

### Gene Expression Analysis

**Figure 7:**
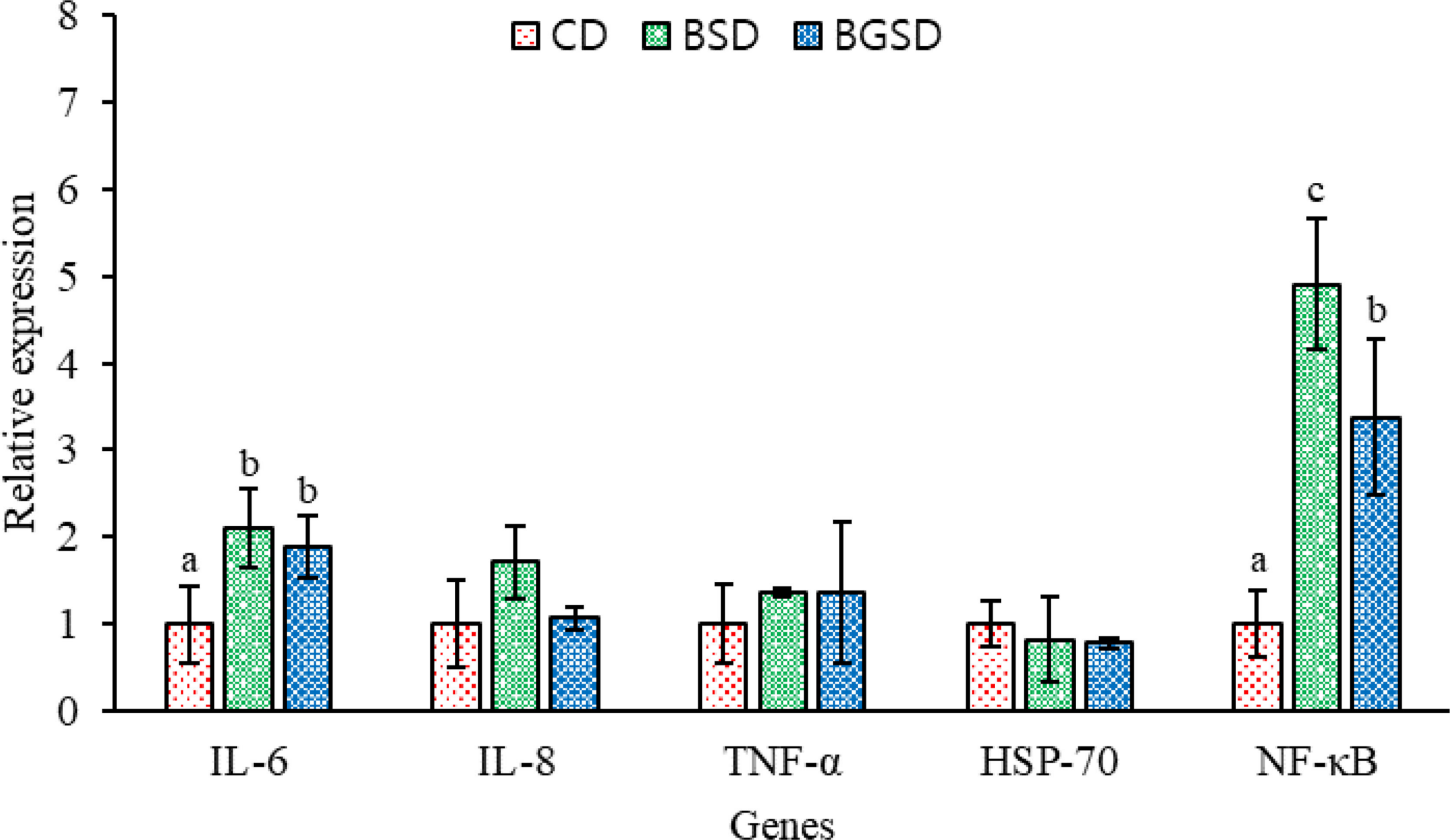
Profiles of immune-related gene expression in the intestine of red sea bream. The expression of these genes was measured in the CD, BSD, and BGSD groups by RT-qPCR after 8 weeks of feeding. The gene expression levels were quantified relative to β-actin transcription. The data are represented as the means ± standard deviation; different letters indicate significant differences between groups (*P* < 0.05). Control diet, CD: without *Bacillus* sp. PM8313 or β-glucan, BSD: 1 × 10^8^ CFU g^−1^ *Bacillus* sp. PM8313, and BGSD: BSD + 0.1% β-glucan.

The dietary additives significantly increased the expression of the immune-related genes IL-6 and NF-κB. However, no significant differences in IL-8, TNF-α, and HSP-70 expression were observed among the experimental groups (**Figure 7**).

### *E. tarda* Challenge

**Figure 8:**
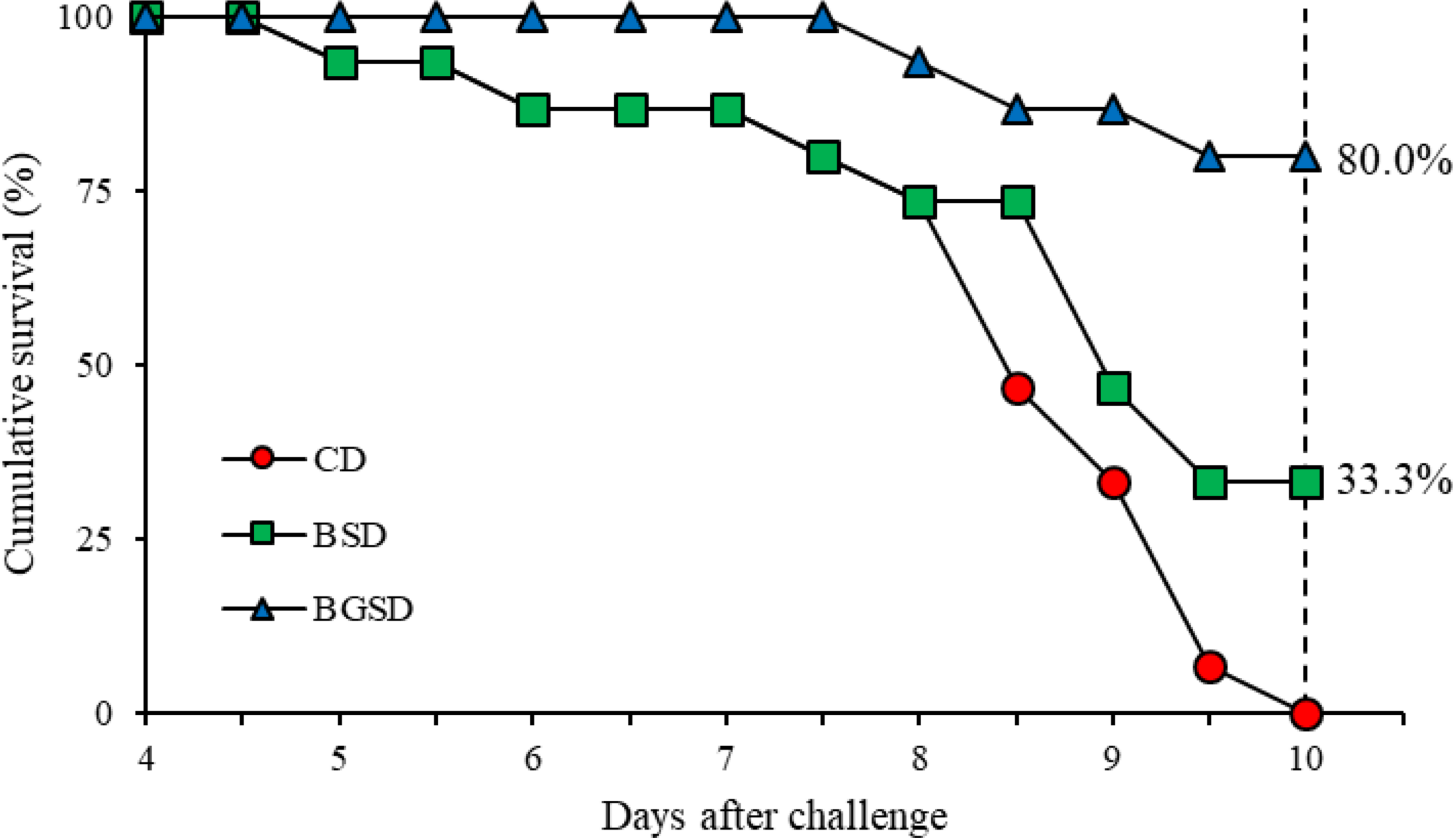
Cumulative survival rates of red sea bream (CD, BSD, and BGSD) challenged with *Edwardsiella tarda* injection (1 × 10^8^ CFU/mL). Means (15 fish/group) were compared at identical times. Different letters indicate significant differences between groups (P < 0.05). Control diet, CD: without *Bacillus* sp. PM8313 or β-glucan, BSD: 1 × 10^8^ CFU g^−1^ *Bacillus* sp. PM8313, and BGSD: BSD + 0.1% β-glucan.

The cumulative survival of red sea bream challenged with *E. tarda* is shown in **Figure 8**. The first mortality occurred five days after intraperitoneal injection. The highest survival rate was observed in the BGSD group. After 10 days of challenge, all fish in the CD group died, and the survival rates of the BSD and BGSD groups were 33.33% and 80.00%, respectively.

## Discussion

Bacteria isolated from the host’s gastrointestinal tract that positively affect the growth and health of the host are referred to as host-associated probiotics (HAPs) (12). Because they adapt to the host defense system and produce many beneficial substances such as digestive enzymes and bioactive compounds, they may be more suitable for probiotic development than bacteria isolated from other sources (12, 26–29). Numerous studies have demonstrated the ability of probiotics to enhance the growth performance and immune response of fish, as well as to modulate the gut microbiota. However, the development of HAPs began only recently in 2020, and therefore very few studies have evaluated their applicability as aquaculture probiotics. Thus, the present study sought to evaluate the effects of dietary supplementation of HAPs on the growth, immunity, and gut microbiota composition of red sea bream.

Positive changes in growth performance and feed utilization were observed in the group fed with strain PM8313, which was isolated from the intestines of healthy red sea bream, both alone or mixed with β-glucan. These results are consistent with previous studies in which probiotic supplementation significantly increased the growth performance of various aquaculture fish species (30–32). Dawood etal. (16) suggested that probiotic supplementation could affect feed palatability, resulting in significant improvement in feed intake and feed utilization parameters (PER, FCR, and protein gain) in fish, thus slightly increasing growth rates in fish. Probiotics may also stimulate the host’s digestive enzymes, thereby enhancing nutrient assimilation (33), or improve nutrient absorption and utilization by improving gut microbiome balance (34). However, no synergistic effects of PM8313 and β-glucan supplementation on growth performance were observed in this study. This may be due to the dosage and frequency of administration, and therefore additional studies are required to maximize the effectiveness of these feed additives.

In the present study, the results of digestive enzyme activity in the anterior midgut of fish further supported the hypothesis that probiotic supplementation may increase digestive enzyme activity, thereby improving growth performance. Supplementation with PM8313 significantly increased amylase, trypsin, and lipase activities. *Bacillus* species are known to secrete digestive enzymes such as amylase, protease, and lipase. The hydrolysis of enzymes secreted by bacteria has been shown to improve the bioavailability of dry matter, proteins, and lipids (35), which may lead to higher growth and nutrient utilization in the host, as shown in this study. The higher digestive enzyme activities observed in the probiotic-supplemented fish were mainly due to the stimulation by the probiotic itself or by exogenous enzymes that promoted the synthesis of endogenous digestive enzymes. In turn, this might have improved nutrient digestibility, resulting in better growth performance and feed efficiency in fish (16). Similarly, previous studies have reported that dietary supplementation with probiotics increases the activity of intestinal digestive enzymes in fish (8, 33, 36);

A growing body of evidence has indicated that probiotics can effectively improve the host’s innate and adaptive immune responses (17). The interaction between host intestinal epithelial cells and probiotics stimulates cellular and humoral immune functions to control the physical and immunological barrier properties of the gut (17, 37). Our study identified significant differences in non-specific immune responses, including SOD and lysozyme activity. SOD is an enzyme that catalyzes the conversion of highly reactive superoxides (i.e., potentially harmful oxygen molecules in cells) into oxygen and hydrogen peroxide to maintain immune homeostasis and prevent tissue damage (38). Lysozyme is found in fish mucus, serum, and tissues where leukocytes exist, and is an important lytic protein in the non-specific defense system that breaks down the cell wall of Gram-positive bacteria (39). PM8313 and β-glucan supplementation significantly increased SOD and lysozyme activity compared to the control group. Similarly, Zaineldin etal. (17) reported that fish fed with a diet supplemented with Bacillus exhibited an increase in non-specific immune responses including serum peroxidase and lysozyme.

After administration, probiotics interact with the intestinal microbial community and block the adhesion of pathogens in the intestinal wall. Probiotic cell wall components like flagella, lipopolysaccharides, and peptidoglycan as well as bacterial nucleic acid are commonly known as microbial-associated molecular patterns (MAMPs). These MAMPs bind with the pathogen pattern receptor (PPRs) of dendritic cells (DCs) or toll-like receptors (TRLs) of enterocytes (40). DCs stimulate phagocytic cells, and activate T and plasma cells to search, engulf and destroy pathogens. TRLs induce the transcription of pro-inflammatory cytokine and production of antibody and antimicrobial peptides from plasma cells and probiotics, respectively involved in the killing and eradication of foreign invaders (37, 41). Although the activation of immune-physiological pathways by PM8313 was not studied, however, obtained results demonstrate the probiotic potential of this bacteria in sea bream.

The gut microbiota of fish is altered by a variety of factors such as habitat, water quality, growth stage, and feeding activity (38, 42). These changes affect fish metabolism, which in turn affects nutrient absorption, metabolic pathways, and ultimately growth (36, 43, 44). Therefore, probiotic supplements can be an excellent strategy to increase fish growth and immunity by regulating the gut microbiota, thus increasing aquaculture yields (38). In this study, PM8313 supplementation induced clear changes in gut microbiota composition. The group fed with PM8313 exhibited a marked decrease in microbial diversity. However, lactic acid bacteria (LAB) such as *Lactobacillus* and *Lactococcus* and other microorganisms became established in the gut microflora of red sea bream. The majority of currently used probiotics are based on LAB strains, and the effects of these probiotics have been demonstrated in various fish species (45). LAB produce antimicrobial compounds such as nisin and pediocin and directly inhibit the growth of Gram-positive pathogenic bacteria (4). Moreover, there was a decrease in the abundance of the fish pathogenic bacteria *Flavobacterium*. Nevertheless, additional studies are needed to elucidate the mechanisms through which the gut microbiota affects the growth and immune response of red sea bream. Particularly, future studies should focus on the changes in the abundance of different (both increases and decreases) due to PM8313 supplementation.

Cytokines are protein mediators produced by immune cells that contribute to the host’s cell growth, differentiation, and defense mechanisms (46). Probiotics directly or indirectly interact with the host’s immune cells to regulate the transcription of genes that play important roles in the immune system, including cytokines (3). In this study, significant differences in IL-6 and NF-κB expression were observed between the intestinal tissues of the additive-supplemented group and the control group. IL-6 is a pleiotropic cytokine that plays an important role in immune homeostatic processes such as inflammation, antibody production by B cells, T cell cytotoxicity, and stem cell differentiation (47). IL-6 is known to stimulate macrophage proliferation and antimicrobial peptide expression in rainbow trout (Oncorhynchus mykiss), as well as the expression of transcription factors that regulate T cell differentiation and antibody production in orange spot grouper (Epinephelus coioides) (48, 49). NF-κB is a key regulator of innate and acquired immune and inflammatory responses and plays an important role in maintaining immune balance in fish (38). Our results demonstrated that PM8313 supplementation improves immunity by regulating the immune-related genes IL-6 and NF-κB in red sea bream, which enhanced the resistance of red sea bream against E. tarda infection.

Collectively, our findings demonstrated the potential of PM8313 as a HAP in aquaculture. Dietary supplementation with PM8313 was found to improve growth performance and digestive enzyme activities, in addition to increasing non-specific immune activity, regulating intestinal microbiota, and increasing resistance to pathogenic strains. This newly isolated HAP could thus be used as a feed additive in red sea bream aquaculture to increase yields and control disease outbreaks.

## Data Availability Statement

The datasets presented in this study can be found in online repositories. The names of the repository/repositories and accession number(s) can be found below: EBI Metagenomics, PRJEB53722; NCBI, PRJNA856430.

## Ethics Statement

The animal study was reviewed and approved by Dong-eui University.

## Author Contributions

WJJ: Conceptualization, Methodology, Software, Investigation, Data Curation, Writing – Original Draft. MHJ: Methodology, Software, Investigation. SJL, SYP, YSL, and DIN: Investigation. SWH, SL, BJL, JML, and MTH: Resources, Data Curation. KWK and EWL: Supervision, Project administration.

## Funding

This work was financially supported by the grant (R2022016) from the National Institute of Fisheries Science, Republic of Korea.

## Conflict of Interest

The authors declare that the research was conducted in the absence of any commercial or financial relationships that could be construed as a potential conflict of interest.

## Publisher’s Note

All claims expressed in this article are solely those of the authors and do not necessarily represent those of their affiliated organizations, or those of the publisher, the editors and the reviewers. Any product that may be evaluated in this article, or claim that may be made by its manufacturer, is not guaranteed or endorsed by the publisher.

## Supplementary Material

Supplementary Figure 1 Phylogenetic tree and genome circular map of *Bacillus* sp. PM8313. **(A)** The phylogenetic tree was constructed by the neighbor joining method using MEGA 7 with 1,000 bootstrap repetitions, after which the 16S rRNA sequences were aligned by the Clustal W program. **(B)** Genome circular map was constructed using GView server. From the center to the outside: the number of bases, GC content, GC skew, location of all annotated ORFs (colored by COG categories).
