# Supplementary material for: Dietary Supplementation of Bacillus sp. PM8313 with β-glucan Modulates the Intestinal Microbiota of Red Sea Bream (Pagrus major) to Increase Growth, Immunity, and Disease Resistance
Source: Front Immunol. 2022 Jul 22;13:960554. doi: 10.3389/fimmu.2022.960554 (PMC9353131; doi:10.3389/fimmu.2022.960554)
Supplement: Supplementary Figure 1 — Phylogenetic tree and genome circular map of Bacillus sp. PM8313. (A) The phylogenetic tree was constructed by the neighbor joining method using MEGA 7 with 1,000 bootstrap repetitions, after which the 16S rRNA sequences were aligned by the Clustal W program. (B) Genome circular map was constructed using GView server. From the center to the outside: the number of bases, GC content, GC skew, location of all annotated ORFs (colored by COG categories). [file Image_1.pdf]

Supplementary Material

1.1 Supplementary Figures

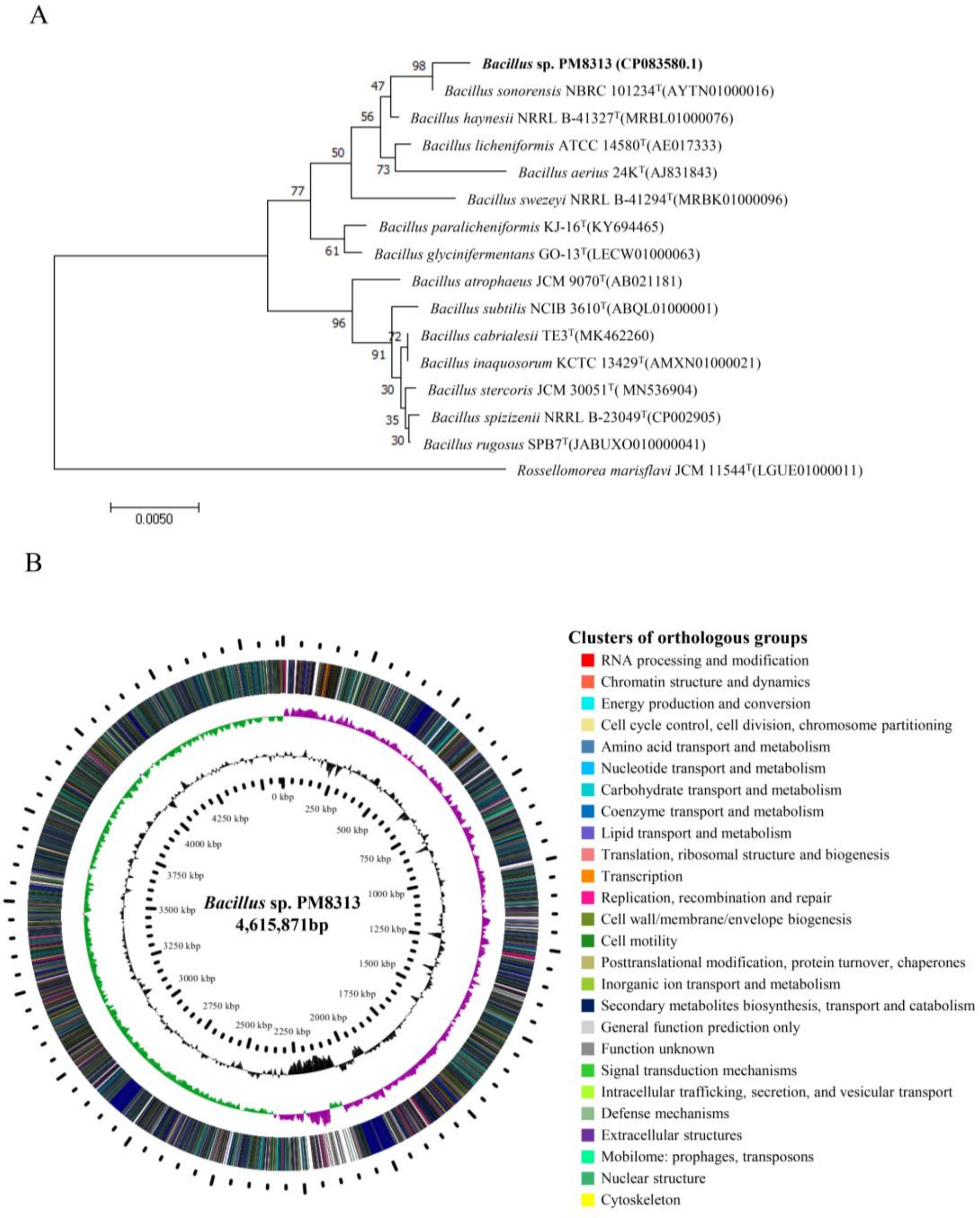

Supplementary Figure 1. Phylogenetic tree and genome circular map of Bacillus sp. PM8313. (A) The phylogenetic tree was constructed by the neighbor joining method using MEGA 7 with 1,000 bootstrap repetitions, after which the 16S rRNA sequences were aligned by the Clustal W program. (B) Genome circular map was constructed using GView server. From the center to the outside: the number of bases, GC content, GC skew, location of all annotated ORFs (colored by COG categories).
